# An effective encoding scheme of obtaining radial topology structures in distribution networks

**DOI:** 10.1186/s40064-016-3104-6

**Published:** 2016-08-31

**Authors:** Juan Wen, Yanghong Tan, Jianmin Zhang

**Affiliations:** 1 College of Electrical and Information Engineering, Hunan University, Changsha, 410082 Hunan People’s Republic of China; 2 College of Electrical and Information Engineering, Nanhua University, Hengyang, 421000 Hunan People’s Republic of China; 3 School of Automation, Hangzhou Dianzi University, Hangzhou, 310018 Zhejiang People’s Republic of China

**Keywords:** Encoding scheme, Loop-switch matrix, Radial topology structure, Distribution network

## Abstract

The structure of a distribution network has great effects on economy, power supply reliability and investment of a power system. To obtain an optimal topology from possible topologies, we need to solve an optimisation problem which aims to find a radial structure satisfying operating constraints. As a basis of solving this optimisation problem, the encoding scheme, is to represent the candidate configurations by a series of codes. Numerical candidate topologies and unfeasible codes would lead low efficiency or premature convergence. This paper presents an effective scheme which can rapidly produce all radial configurations of a distribution network. In order to reduce the computational requirement of solution space, initial network is simplified as a topological graph which reserves loop branches and T-nodes. And a loop-branch chain incidence matrix is derived from analyzing the relationship between any two loops. Then the principles of selecting switches of each variable are designed to determine the ranges of the variables. All radial candidate solutions are available rapidly through applying the theory of combination. The scheme presented minimizes the number of solutions and avoids tedious radial checking procedure in view of avoiding any infeasible solutions. The validity of the proposed scheme is verified by illustrative examples.

## Background

Distribution network topological structure plays a vital role in improving the reliability and efficiency of power system (Narimani et al. 2014). Distribution feeders are connected in a radial structure that is maintained by appropriately altering the status of the switches installed on the distribution networks. There are two types of switches, that is, sectionalizing switches and tie switches. The sectionalizing switches are normally closed switches on connecting the line sections and the tie switches are normally open switches on the tie-lines connecting either two primary feeders. By changing the status of some switches, loads can be transferred from one feeder to an adjacent one to generate a reconfigured network topology. Thus, the switching combinations determine the reconfigured network structures in a distribution system. There are many possible candidate topologies in a distribution network because of the large number of candidate switching combinations. It is a complicated combinatorial optimisation problem to obtain an optimal topology from these candidate topologies. This optimisation aims at finding a radial structure that satisfies operating constraints. The artificial intelligence algorithms have widely used to address the optimisation problem recently (Tang et al. 2014; Rao et al. 2013; Hu et al. 2014; Tolabi et al. 2011; Gupta et al. 2014; Alonso et al. 2015; Golshannavaz et al. 2014). An encoding scheme, as the base of the intelligence optimisation approaches, is to represent the candidate configurations by a series of decimal codes.

The control variables of the encoding scheme are sectionalizing switches and tie switches, which are expressed as a set of decimal number. The encoding space depends on the number of possible solutions generated from coding scheme. The solution space is composed of all possible combinations of control variables. The codes of feasible solutions in solution space are named valid codes and the corresponding topologies satisfy the constraints of network reconfiguration. The size of the solution space will be increased as the increasing of candidate solutions. Moreover, the large scale of solution space would be disturbed the optimisation process if one optimal solution is identified from numerical possible solutions. Furthermore, the validity of solutions ensures that a meaningful new solution in each generation is obtained by combining original codes. Evidently, a good encoding scheme has a significant influence on the efficiency and convergence time of optimisation.

Various encoding schemes are proposed to solve the optimisation problem (Sivanagaraju et al. 2008; Fontan 2008; Zhu 2002; Sawa 2009; Andervazh et al. 2013; Wu and Tsai 2011; Asrari et al. 2015; Jikeng et al. 2013; de Macedo Braz and de Souza 2011; Santos et al. 2010). An attempt to express all switches as a set of configuration variables is presented in Sivanagaraju et al. (2008). Each parameter of solution vector represents a switch. Since the value 0 and 1 denotes that a switch is open or closed, it is named binary coding. Fontan (2008) has proposed an improved characteristic vector which the network topology string stores only the opened switches positions. An encoding strategy based on keeping the number of chords constant has presented in Zhu (2002). Sawa (2009), Andervazh et al. (2013) and Wu and Tsai (2011) employ a decimal integer loop encoding strategy which only one switch is opened in each fundamental loop. A look-up table based on loop cut set is built and each loop is considered as a control variable. The number of each open switch is lined up with the order of the loop number. One group of decimal integers is uniquely corresponding to a candidate solution. Based on the integer loop encoding strategy, a reliability-based encoding scheme is presented in Asrari et al. (2015). For a distribution system with *m* switches and *n* tie switches, the possible solutions in Sivanagaraju et al. (2008), Fontan (2008), Zhu (2002) and Sawa (2009) are $2^{m} ,\, C_{m - l }^{n} ,\,m^{n}$ and $\prod\nolimits_{i}^{n} {m_{i} } ,$ where $m_{i}$ is the number of switches in *i*th fundamental loop and *l* is the number of switches outside loops. For example, in a 33-bus system with m = 37 and n = 5, the number of candidate switching combinations in Sivanagaraju et al. (2008), Fontan (2008), Zhu (2002) and Sawa (2009) are 1.3744 × 10^11^, 376,992, 69,343,957 and 242,550, respectively. And the proportion of feasible solutions is only 0.00004, 13.46, 0.0732 and 20.92 %. The computational requirement of these schemes is considerable and they require a tedious radial checking procedure to remove the infeasible solutions. As a result, the performance of optimisation methods is dramatically reduced because the validation of the solutions usually is time consuming. Jikeng et al. (2013) proposes a method using basic tree to obtain the radial topology of the distribution network. The sequential encoding and node-depth encoding are presented in de Macedo Braz and de Souza (2011) and Santos et al. (2010). Although the candidate solutions generated by these approaches are all valid codes, the encoding scheme is complex as they demand optimisation algorithms with dynamic parameters. Furthermore, these schemes in (de Macedo Braz and de Souza 2011; Santos et al. 2010) cannot be used to calculate the feasible solutions in view of their large size of the solution spaces.

This paper proposes an effective encoding strategy which mitigates the computational burden and obtains rapidly all candidate configurations in a distribution network. The initial network is simplified as a topological graph which only reserves T-nodes of loops and corresponding branch chains. A loop-branch chain matrix is established according to the relationship between any two loops of simplified network. Combining with the designed principles of selecting branch variables, all possible solutions that satisfy constraint conditions are obtained by fixing the diagonal element of the matrix. All the unfeasible solutions can be completely avoided, thus it leads to dramatic reduction of the size of solution space. Numerical experiment was executed to evaluate the proposed encoding scheme in comparison with the methods (Sivanagaraju et al. 2008; Fontan 2008; Zhu 2002; Sawa 2009; Asrari et al. 2015; Jikeng et al. 2013; de Macedo Braz and de Souza 2011).

The next section introduces the preprocessed network model and the nomenclature to be used in the following sections. The paper continues proposing encoding criteria and the effective encoding scheme description. We applied the scheme to three test systems and show the results in “Case study” section.

## Network modeling and simplification

**Fig. 1:**
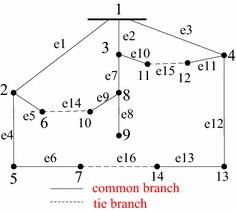
Example network topology

In graph theory viewpoint, a graph is made up of vertices and edges. Vertices act as nodes within the graph. And edges act as branches which connect vertices together (Santos et al. 2010). A distribution system is described as a set of bus bars connected together by switches, then bus bars would become nodes and the switches would become branches. Consider a system with *n* buses and *m* switches, it can be modeled as an abstract graph with *n* nodes and *m* branches. The graph is denoted as $G = \left( {V,E} \right),$ where $V = \left\{ {v_{1} ,v_{2} , \ldots v_{n} } \right\}$ is a set of labeled nodes and $E = \left\{ {e_{1} ,e_{2} , \ldots e_{m} } \right\}$ is a set of branches. Due to the switches are divided into sectionalizing switches and tie switches, the corresponding branches are named as common branches and tie branches. A diagrammatically representation of a distribution network is illustrated, using example network as shown in Fig. 1. Branches in dotted connecting nodes (6–10), (11–12), and (7–14) are tie branches, and other branches are common branches. The topology network is reconstructed by opening/closing of these branches.

An adjacency matrix of network topology is defined as $A = \left( {a_{ij} } \right)_{n \times n} ,$ where *n* is the number of nodes, $a_{ij}$ is a binary variable, if $a_{ij} = 1$ demonstrates *i*-node is connected to another *j*-node by a branch. Otherwise, $a_{ij} = 0.$ The degree of *i*-node is defined as $D_{vi} = \sum\nolimits_{j = 1}^{n} {a_{ij} }$. Generally a distribution network incorporates a few kinds of nodes, i.e. terminal nodes, electric T-nodes and junction nodes. So the different sets of degree value $D_{vi} = 1,\,D_{vi} = 2$ and $D_{vi} \ge 3$ are corresponding to the terminal nodes, junction nodes and T-nodes.

**Fig. 2:**
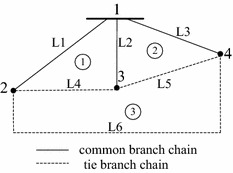
Simplified graph of example system

Due to there are a large number of branches in the real distribution network, the codes would be long and redundancy. It is necessary to simplify the network for avoiding the invalid solutions and reducing the code length. The loops would appear if all the branches are closed in the distribution network. Some branches which don’t belong to any loop could be omitted in coding. Only the loop branches are taken into consideration as the control variables in this encoding strategy. And the branches between any two T-nodes could be merged into a branch-group. So the initial topology is simplified a graph which incorporates T-nodes and corresponding branch chains. Figure 2 is the simplified generation from Fig. 1. The number of nodes and branch chains in the simplified graph are 4 and 6, respectively. The branch chains of including tie branches are called tie branch chains and others are common branch chains.

## Effective encoding scheme

**Fig. 3:**
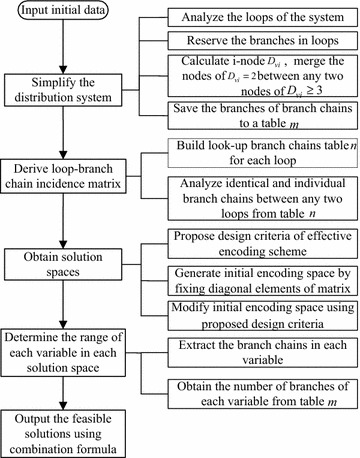
The flowchart of the proposed encoding scheme

Based on the simplified topology graph of reserving the loop branches and T-nodes, an effective encoding scheme of obtaining radial topologies is formulated. The number of dimensions of each solution is equal to the number of loops in the network. All loop branches are divided into different groups and each group is coded in one dimension of a solution. Observing the relationship of between any two loops, a loop-branch chain matrix is derived. The principles of choosing branch chains of each solution are designed. According to these principles, the encoding space is determined by fixing diagonal parameters of the matrix based. And the upper bounds of any variable are obtained through extracting the branches from each branch chains. The lower bounds are equal to 1. The flowchart of the proposed scheme is shown in Fig. 3.

As shown in Fig. 2 as an example system, a detailed description of implementing the encoding scheme. Previous works are based on fundamental loops vectors that only one tie branch of each loop is opened (Sawa 2009; Andervazh et al. 2013; Wu and Tsai 2011). In this section, a novel approach for determining the loops is introduced. It is required that there is minimum number of branch chains and maximum number of tie branch chains in each loop. The number of loops is still equal to tie branch chains. A serial number is assigned to branch chains of each loop. The branch chains in each loop for Fig. 2 are shown in following Table 1.

**Table 1:** Look-up branch chains table for each loop

Loop	Branch chains
Loop ①	L1 L2 L4
Loop ②	L2 L3 L5
Loop ③	L4 L5 L6

We are interested in analyzing the relationship between any two loops of the network. It can be described as a particular loop-branch chain matrix which has one row for each loop and one column for each the set of branch chains. The incidence matrix can be formulated as Eq. (1).

1 $$L = (l_{ij} )_{n \times n} = \left[ {\begin{array}{*{20}l} {L1} \hfill &\quad {L2} \hfill &\quad {L4} \hfill \\ {L2} \hfill &\quad {L3} \hfill &\quad {L5} \hfill \\ {L4} \hfill & \quad {L5} \hfill &\quad {L6} \hfill \\ \end{array} } \right]$$

where *L* is loop-branch chain matrix, *n* is the number of loops, $l_{ij}$ is a variable, if $l_{ij } = L_{x} \left( {L_{x} \in \left( {L1\,\sim\,L6} \right)} \right)$ demonstrates there is a public branch chain $L_{x}$ between loop *i* and loop *j*, otherwise, $l_{ij } = 0.$

For a simplified network with *m* branch chains and *n* loops, the dimension of *L* matrix is *n* row and *n* column. The diagonal parameters appear only once in the matrix, which represents the corresponding branch chains only exist in one loop. Other elements are common branch chains between the two loops. Thus, this matrix is symmetric.

A possible solution of a distribution network has a unique code in encoding scheme. Redundant codes will lead inefficiency and premature convergence during the searching process as it needs to identify one feasible solution from numerical candidate solutions. Invalid codes may be produce meaningless solutions. To reach any point in solution space, an ideal encoding scheme has characteristics of non-redundancy, validity and completeness. On the basis of loop-branch chain incidence matrix, we propose the following criteria which are used to obtain effective candidate solutions.

1. The number of opened branches is equal to the number of independent loops in a distribution network.
2. Diagonal set of branch chains should be at least one opened branch. It is important to prevent the emergence of loop structure.
3. For avoiding the isolated nodes, only one branch is opened in the same set of branch chains.
4. There must be at least one opened branch in each column of *L* matrix.
5. Each branch chain could not be repeated in a set of branches.

According to the first to fourth principles, the size of initial solution space is determined by the dimension of matrix *L*. Each loop is considered as one control variable and one diagonal parameter of loop-branch chain matrix must be chosen. It means that the corresponding loop label of this parameter row label is fixed. Other opened branches are obtained by applying the other criteria. If a diagonal branch chain is once selected, it will be removed. That is, this branch chain is omitted in the formation of other solution spaces. Consider a loop-branch chain matrix of Fig. 2, the initial solution spaces are described as Table 2.

**Table 2:** Initial encoding space of example network

Fixed loop	Encoding form
Loop ①	Subspace 1: [{L1} {L2 L3 L5} {L4 L5 L6}]
Loop ②	Subspace 2: [{L2 L4} {L3} {L4 L5 L6}]
Loop ③	Subspace 3: [{L2 L4} {L2 L5} {L6}]

The initial solution space has three subspaces and shown in Table 2. Each loop has only one opened branch chain in each subspace. It may be produce invalid codes because a switch must be a member of loops more than once. For example, the branch chain collection on each loop of subspace 1 is {L1}, {L2 L3 L5} and {L4 L5 L6}, respectively. The L5-branch chain is a member of both loop ➁ and loop ➂. An unexpected solution of [{L1} {L5} {L5}] may be appeared. To address this problem, the solution space needs to be divided again according the principle (v). Therefore, this coding strategy has no redundancy coding and all the trial solutions are satisfied topology constraints for the network. The initial solution spaces are divided into multiple subspaces (sp1–sp6) and the loop combination branches of each subspace are described as Table 3.

**Table 3:** Loop constructed branches

Subspace number	Encoding scheme	Constructed branches		
		Loop ①	Loop ②	Loop ③
Subspace 1	[{L1} {L2 L3 L5} {L4 L6}]	e1	e2, e3, e10, e15, e11	e5, e14, e9, e7, e4, e6, e16, e13, e12
Subspace 2	[{L1} {L2 L3} {L5}]	e1	e2, e3	e10, e15, e11
Subspace 3	[{L2 L4} {L3} {L5 L6}]	e2, e5, e14, e9, e7	e3	e10, e15, e11, e4, e6, e16, e13, e12
Subspace 4	[{L2} {L3} {L4}]	e2	e3	e5, e14, e9, e7
Subspace 5	[{L2 L4} {L5} {L6}]	e2, e5, e14, e9, e7	e10, e15, e11	e4, e6, e16, e13, e12
Subspace 6	[{L4} {L2} {L6}]	e5, e14, e9, e7	e2	e4, e6, e16, e13, e12

A set of solutions is formed through extracting a random branch each loop in the subspace. For example, the number of solution combinations in subspace 1 is $C_{1}^{1} \times C_{5}^{1} \times C_{9}^{1} = 45.$ The solution combinations of subspace 2 through subspace 6 have 6, 40, 4, 75 and 20, respectively. It is obviously that all the constructed branches of each subspace are not duplicated. The sum of solution combinations is equal to the number of radial topologies in the system. Therefore, the trial solutions are validity and non-redundancy.

## Cases study

The performance of the proposed encoding scheme is demonstrated by testing on 16-bus, 33-bus and 69-bus radial distribution systems. For all these systems, all tie and sectionalizing branches are normal. The experimental results are obtained to evaluate its effectiveness. All scenarios have been programmed in MATLAB software. The simulations are implemented on a computer with the processor of Intel core i7 CPU and windows 10 operating system.

### Test case 1

The first test case is a 16-bus distribution system and the structure is presented in Werho et al. (2016). It consists of 14 nodes, 13 sectionalizing switches and 3 tie switches. The normally closed switches are 1–13, and normally open switches are 14–16. The undirected graph after closing normally open branches is depicted in Fig. 1. The simplified topological graph and loop-branch chain incidence matrix are described as Fig. 2 and Eq. (1). The program running time and the size of solution space are considered as the parameters to analyze the performance of the coding strategy. The calculation results are summarized and compared to encoding methods presented in Sivanagaraju et al. (2008), Fontan (2008), Zhu (2002), Sawa (2009), Asrari et al. (2015), Jikeng et al. (2013) and de Macedo Braz and de Souza (2011). Table 4 shows the average run time of obtaining the first valid solution and all feasible solutions for above mentioned methods.

**Table 4:** Computational results of 16-bus system

Method	Solution space	The ratio of feasible solution (%)	Run time of obtaining radial solutions (s)	The time of obtaining the first feasible solution (s)
Sivanagaraju et al. (2008)	2^16^ = 65,536	0.29	17.7024	0.172562
Fontan (2008)	$C_{15}^{3}$ = 455	41.76	0.1954	0.006554
Zhu (2002)	16^3^ = 4096	4.64	0.8145	0.078451
Sawa (2009)	7 × 6 × 5 = 210	90.48	0.0943	0.002125
Asrari et al. (2015)	210	90.48	0.0930	0.002037
Jikeng et al. (2013)	190	100	0.0917	0.001078
de Macedo Braz and de Souza (2011)	–	–	–	0.000990
Proposed scheme	190	100	0.0826	0.000945

–, represents no mention

For this system, almost all the schemes can get a solution space except to sequential encoding of de Macedo Braz and de Souza (2011). The number of radial configurations is given by using basic tree search method (Jikeng et al. 2013). The size of solution space by the proposed scheme is 190, which is consistent with (Jikeng et al. 2013) but less than other schemes (Sivanagaraju et al. 2008; Fontan 2008; Zhu 2002; Sawa 2009). Due to a large number of solutions, the ratio of feasible solution in Sivanagaraju et al. (2008), Fontan (2008) and Zhu (2002) is 0.29, 41.76 and 4.64 %, respectively. Although the methods of Sawa (2009) and Asrari et al. (2015) are improved to 90.48 %, some unfeasible codes still exist in the solution space. So the number of analyzing solutions’ validity computer memory required for the proposed method is less than method in Sivanagaraju et al. (2008), Fontan (2008), Zhu (2002), Sawa (2009) and Asrari et al. (2015). To obtain the radial solutions, the branch-to-node incidence matrix method is used to check the radiality and remove the infeasible solutions in this paper (Alonso et al. 2015). The least time identified the first feasible solution by referenced methods is 0.000990 s (de Macedo Braz and de Souza 2011). Our method is able to find the first radial configuration in 0.000945 s which is less than de Macedo Braz and de Souza (2011). The average run time of obtaining all radial solutions for the schemes are 17.7024, 0.1954, 0.8145, 0.0943, 0.8145 0.0930, 0.0917 and 0.0826 s, respectively. It illustrates the our encoding strategy requires less time to get all feasible solutions if the basic tree search method obtained valid codes are the same. The results presented demonstrate that the proposed scheme is much faster and more efficient than those by conventional methods. Hence, this scheme is able to present more benefits to intelligent reconfiguration algorithms.

### Test case 2

**Fig. 4:**
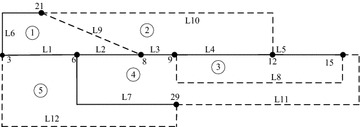
Simplified graph of 33-bus system

The next case system is a hypothetical electrical distribution system with 33 buses and five loops. Andervazh et al. (2013) gives the system data of initial configuration. Following the steps presented in “Network modeling and simplification” section, a simplified topology structure is shown in Fig. 4. The number of nodes is 8 instead of 33 and the number of branches is 12 instead of 37. The relationship between any two loops can be described as expression (2). To verify the effectiveness of the proposed scheme, Table 5 provides the responses obtained with the encoding schemes tested for the 33-bus system.

2 $$L = (l_{ij} )_{m \times m} = \left[ {\begin{array}{*{20}l} {L6} \hfill &\quad {L9} \hfill &\quad 0 \hfill &\quad {L1} \hfill &\quad {L2} \hfill \\ {L9} \hfill &\quad {L10} \hfill &\quad {L4} \hfill &\quad 0 \hfill &\quad {L3} \hfill \\ 0 \hfill &\quad {L4} \hfill &\quad {L5} \hfill &\quad 0 \hfill &\quad {L8} \hfill \\ {L1} \hfill &\quad 0 \hfill &\quad 0 \hfill &\quad {L12} \hfill &\quad {L7} \hfill \\ {L2} \hfill &\quad {L3} \hfill &\quad {L8} \hfill &\quad {L7} \hfill &\quad {L11} \hfill \\ \end{array} } \right]$$

**Table 5:** Search space of different encodings for systems

Reference	33-bus system				69-bus system				*m*-bus system
	Size of solution space	Feasible solution (%)	Rt1 (s)	Rt2 (s)	Size of solution space	Feasible solution (%)	Rt1	Rt2 (s)	Size of solution space
Sivanagaraju et al. (2008)	2^37^ = 1.3744 × 10^11^	0.00004	–	197.04	2^73^ = 5.9030 × 10^20^	3.4714 × 10^−15^	–	1018.34	2 ^*m*^
Fontan (2008)	${\text{C}}_{ 3 6}^{ 5}$ = 376,992	13.46	465.94	2.5131	${\text{C}}_{ 7 1}^{ 5}$ = 4,187,106	7.83	–	2.6507	$C_{m }^{n}$
Zhu (2002)	37^5^ = 69,343,957	0.0732	–	125.9735	73^5^ = 2.0731 × 10^9^	0.0158	–	541.0476	*m* ^*n*^
Sawa (2009)	10 × 15 × 7 × 21 × 11 = 242,550	20.92	294.76	0.0154	17 × 8 × 24 × 32 × 17 = 1,775,616	18.47	8.1 h	0.0482	$\prod\nolimits_{i}^{n} {m_{i} }$
Asrari et al. (2015)	242,550	20.92	253.72	0.0143	1,775,616	18.47	7.6 h	0.0445	–
Jikeng et al. (2013)	50,751	100	79.35	0.0049	327,868	100	292.02 s	0.0088	–
de Macedo Braz and de Souza (2011)	–	–	–	0.0032	–	–	–	0.0059	–
Proposed scheme	50,751	100	0.1092	0.0024	327,868	100	0.2068 s	0.0034	$\sum\nolimits_{j = 1}^{j} {\prod\nolimits_{n = 1}^{n} {m_{jn} } }$

-, represents a long time over 12 h to obtain all feasible solutions; –, represents results cannot be calculated; Rt1, represents the average run time of obtaining all radial solutions; Rt2, represents the average run time of obtaining first radial solution

From the Table 5, the solution space for this system is significantly greater than the 16-bus system. Among the references consulted, only the sequential encoding (Jikeng et al. 2013) is unable to calculate the solution space. The minimum solution space obtained by the methods of Sivanagaraju et al. (2008), Fontan (2008), Zhu (2002) and Sawa (2009) is 242,550, but the proportion of feasible solutions is only 20.92 %. It would take a very long time to obtain all feasible solutions and sometimes is impossible. Using the proposed scheme, the number of branch combinations is 50,751, which is the same as the result of Jikeng et al. (2013) but much less than other coding strategies. The least run time consumed to reach the first feasible solution in conventional methods is sequential encoding (Asrari et al. 2015), spending 0.0143 s. And the corresponding time is 0.0024 s in our scheme. Based on the run time of obtaining all feasible solutions, our method is the fastest to achieve the goal with Rt2 = 0.1092 s on average. Therefore, the proposed scheme can identify the first feasible solution and obtain all the valid solutions rapidly.

### Test case 3

**Fig. 5:**
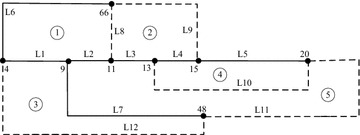
Simplified graph of 69-bus system

This test system is a hypothetical 12.66-kV 69-bus distribution system with five normally open switches. The branches and nodes of initial structure are given in Rao et al. (2013). Figure 5 shows the simplified topology graph and the expression (3) describes the loop-branch chain incidence matrix.

3 $$L = \left[ {\begin{array}{*{20}l} {L6} \hfill &\quad {L8} \hfill &\quad {L1} \hfill &\quad 0 \hfill &\quad {L2} \hfill \\ {L8} \hfill &\quad {L9} \hfill &\quad 0 \hfill &\quad {L4} \hfill &\quad {L3} \hfill \\ {L1} \hfill &\quad 0 \hfill &\quad {L12} \hfill &\quad 0 \hfill &\quad {L7} \hfill \\ 0 \hfill &\quad {L4} \hfill &\quad 0 \hfill &\quad {L5} \hfill &\quad {L10} \hfill \\ {L2} \hfill &\quad {L3} \hfill &\quad {L7} \hfill &\quad {L10} \hfill &\quad {L11} \hfill \\ \end{array} } \right]$$

Table 5 presents the comparison of the proposed scheme and approaches presented in Sivanagaraju et al. (2008), Fontan (2008), Zhu (2002), Sawa (2009), Asrari et al. (2015), Jikeng et al. (2013) and de Macedo Braz and de Souza (2011). Under the viewpoint of quality of encoding, only sequential encoding (Jikeng et al. 2013) is unable to calculate the solution space. Using the mentioned methods in Sivanagaraju et al. (2008), Fontan (2008), Zhu (2002), Sawa (2009) and Asrari et al. (2015), there are many trail solutions because large number of infeasible solutions exist in the solution space. And the ratio of valid codes in entire encoding space is lower than 5 %. It is almost impossible to identify all the possible radial solutions in a short time. The number of feasible solutions obtained by the proposed method is 327,868, which less than above the approaches. The method are presented in Jikeng et al. (2013) can accomplish the searching result of solution space, but the run time of obtaining the all the possible solutions takes 292.02 s. Referring to Table 5, in the proposed scheme, it takes only 0.2068 s to obtain all feasible configurations. Evaluation running time Rt2, our scheme achieves the first feasible solution faster than other methods, i.e. 0.0034 s against 0.0059 s in de Macedo Braz and de Souza (2011), 0.0034 s compared to 0.0088 s in Jikeng et al. (2013), and 0.0034 s instead of 0.0445 s in Asrari et al. (2015).

From the above results, the number of possible solutions of a distribution network is related to the common branches and tie branches. The proposed method is dramatically reduced the number of possible configurations as it avoids the unfeasible solutions. The computational requirement for a network with *m* branches and *n* tie branches is $\sum\nolimits_{j = 1}^{j} {\prod\nolimits_{n = 1}^{n} {m_{jn} } } ,$ where *j* represents the number of subspaces, and $m_{jn}$ is the number of branches in *n*th loop of each subspace. Furthermore, the run time of achieving the first feasible solution by our scheme remains at the forefront. In direct comparison the time (Rt1), an apparent advantage of the proposed scheme is that could obtain the all valid solutions fast, i.e., 0.1092 s in 33-bus system and 0.2068 s in 69-bus system.

## Conclusions

Due to the extremely large number of unfeasible solutions, the size of solution space of traditional encoding schemes is very considerable and would lead time consuming before selecting an optimal solution. This work proposes an effective encoding scheme which can rapidly produce trail solutions of a distribution network. The advantage of this method is only generated the radial configurations. It is possible that the encoding strategy avoids the unfeasible solutions and thus reduces the solution space dramatically. The computational burden may be alleviated as it does not need to verify the validity of the trial solutions. The validation tests are carried on 16-bus, 33-bus and 69-bus systems, and the test results are compared with other conventional schemes. Test results show the proposed encoding scheme can ensure each trial solution to be feasible. Thus, it has been able to get all feasible solutions with a few calculations. And this scheme minimizes significantly the solution space. Furthermore, we can obtain the first feasible solution quickly. In future, it is useful to improve the intelligence reconfiguration algorithms in terms of computational speed and memory requirements.
